# A Syndemic Perspective on the Management of Non-communicable Diseases Amid the COVID-19 Pandemic in Low- and Middle-Income Countries

**DOI:** 10.3389/fpubh.2020.00508

**Published:** 2020-09-25

**Authors:** Uday Narayan Yadav, Binod Rayamajhee, Sabuj Kanti Mistry, Shradha S. Parsekar, Shyam Kumar Mishra

**Affiliations:** ^1^Centre for Primary Health Care and Equity, University of New South Wales (UNSW), Sydney, NSW, Australia; ^2^Forum for Health Research and Development, Dharan, Nepal; ^3^School of Optometry and Vision Science, Faculty of Science, University of New South Wales, Sydney, NSW, Australia; ^4^Department of Infectious Diseases and Immunology, Kathmandu Research Institute for Biological Sciences (KRIBS), Lalitpur, Nepal; ^5^James P Grant School of Public Health, BRAC University, Dhaka, Bangladesh; ^6^Public Health Evidence South Asia, Prasanna School of Public Health, Manipal Academy of Higher Education, Manipal, India; ^7^Department of Microbiology, Institute of Medicine, Tribhuvan University, Kathmandu, Nepal

**Keywords:** SARS-CoV-2, COVID-19, LMICs, NCDS, syndemic framework

## Abstract

The global coronavirus disease (COVID-19) pandemic has greatly affected the lives of people living with non-communicable diseases (PLWNCDs). The health of PLWNCDs worsens when synergistic epidemics or “syndemics” occur due to the interaction between socioecological and biological factors, resulting in adverse outcomes. These interactions can affect the physical, emotional, and social well-being of PLWNCDs. In this paper, we discuss the effects of the COVID-19 syndemic on PLWNCDs, particularly how it has exposed them to NCD risk factors and disrupted essential public health services. We conclude by reflecting on strategies and policies that deal with the COVID-19 syndemic among PLWNCDs in low- and middle-income countries.

## Introduction

The entire world has been affected by the coronavirus disease (COVID-19) pandemic caused by severe acute respiratory syndrome coronavirus 2 (SARS-CoV-2), which has led to thousands of deaths each day. The COVID-19 pandemic is one of the greatest public health calamities since World War II and, despite best efforts, has been challenging to control (1). Recognizing the rapid spread of COVID-19 and the threats it poses, the World Health Organization (WHO) declared it an international public health emergency on 30 January 2020. This allowed countries to exert maximum effort and allot resources to limit the rapid transmission of SARS-CoV-2. Despite the low fatality rate and government efforts, people are living in uncertainty and fear, as there is no vaccine for COVID-19. COVID-19 has weakened healthcare systems and economies, emptied open spaces, and filled hospitals (2). The pandemic has separated many people from their family, friends, and workstations and has severely disrupted modern life.

To mitigate this unprecedented pandemic, physical, and social distancing along with nationwide lockdowns and restrictions, have been implemented for the past few months in several countries (3). COVID-19 is creating a profound impact on all parts of the community, including the physical and mental health of the public. The growing pandemic is augmenting existing mental health problems (4), including loneliness, anxiety, paranoia, panic, depression, and hoarding, with long-term psychosocial impacts (5). Social distancing, stress, and fear are the main factors behind these psychological problems, leading to a global increase in suicides (6). Self-isolation and quarantine measures disproportionately affect people, especially older adults, migrants, laborers, refugees, people with chronic diseases, and marginalized and vulnerable populations (7). The COVID-19 cataclysm has become the most serious problem worldwide, and its consequences have left no one untouched (6).

**Figure 1:**
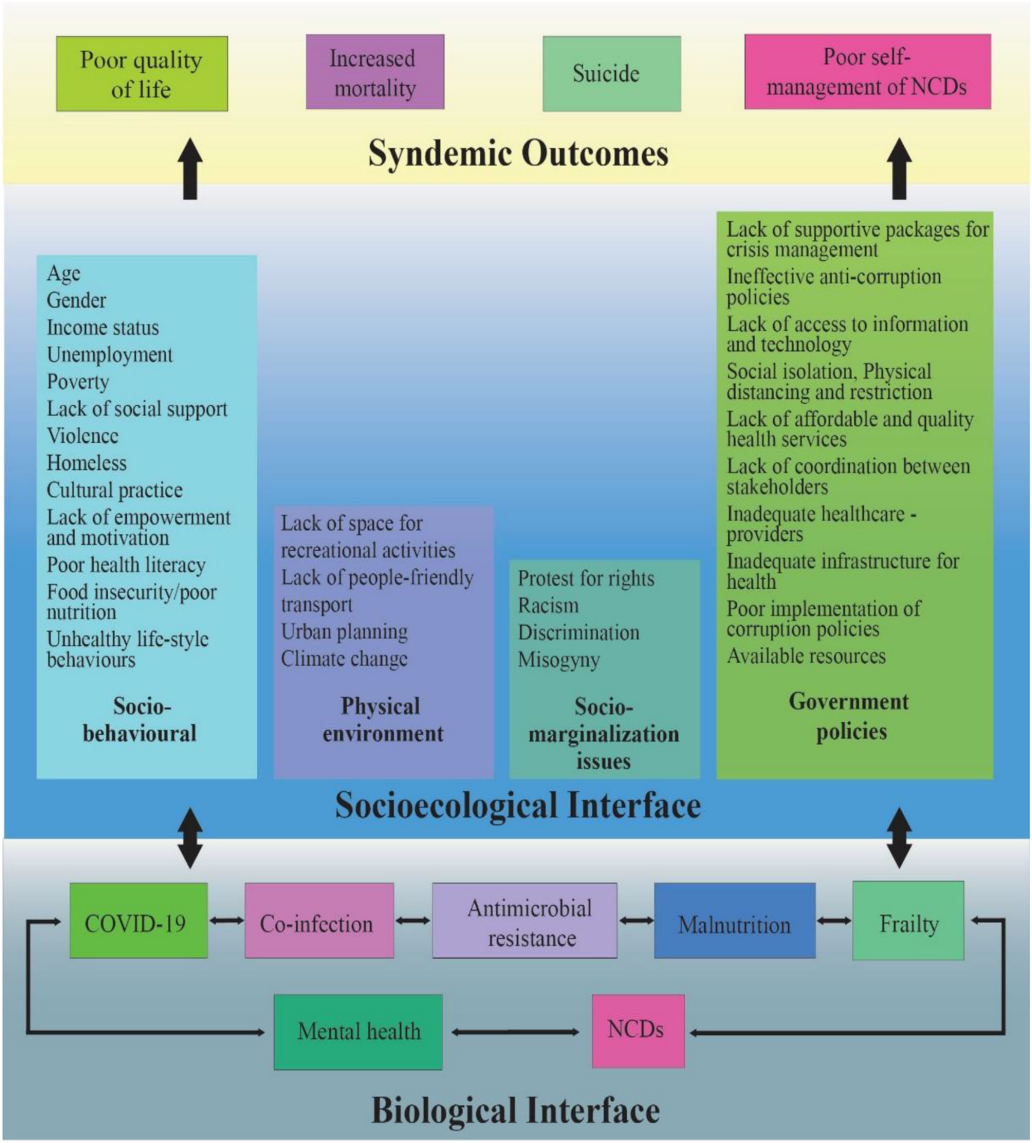
Fueling factors responsible for COVID 19 syndemic outcomes among PLWNCDs [adapted from Singer (8), Bambra (9), and Shaiu (10)].

The effects of a pandemic intensify due to its diverse nexus of intertwined biological and socioecological factors. This diverse nexus was coined a “syndemic” by medical anthropologist Merrill Singer in the 1990s to describe the relationship between HIV/AIDS, substance use, and violence (8). A “syndemic” is defined as a synergistic interaction between socioecological and biological factors (Figure 1), resulting in adverse health outcomes (9). The COVID-19 pandemic has escalated into a syndemic due to several driving factors, such as overcrowding, loneliness, uncertainty, poor nutrition, and lack of access to health services; consequently, depression, suicide, domestic violence, and psychiatric illnesses have significantly increased (11). Social determinants of health, such as poverty, social inequality, social stigma, and the environment where people live and work, greatly affect the intensity of the syndemic (12). Additionally, xenophobia, ostracism, and racism are reported in many places. Generally, people living in countries with higher social and economic inequalities have more coexisting non-communicable diseases (NCDs) and are therefore more vulnerable to the syndemic impact of COVID-19.

We argue that, for people living with NCDs (PLWNCDs), COVID-19 is considered a syndemic—a synergistic pandemic that interacts with various pre-existing medical conditions and social, ecological, and political factors and exacerbates existing NCDs. Studies have reported higher proportions of frailty (13, 14), malnutrition (15), psychological problems (16), and co-infections, including antimicrobial resistance pathogens, among PLWNCDs (17) in low- and middle-income countries (LMICs). NCDs have been recognized as a key risk factor for COVID-19 patients (18); however, vulnerability to catching SARS-CoV-2 increases in the presence of other pre-existing factors. Prevailing inequalities in the social determinants of health, including poor social, economic, and environmental conditions (e.g., social behavioral factors, physical environment, social marginalization, and supportive government policies; Figure 1), have an impact on various aspects of life such as health, wellness, and financial status. For example, PLWNCDs with comorbidities and higher social and economic deprivation are less likely to access health services during this pandemic. This results in worse health outcomes, such as poor quality of life, mortality, suicide (6, 19, 20), and increased hospitalization due to poor self-management (21, 22).

During the COVID-19 pandemic, PLWNCDs from disadvantaged groups are less likely to receive healthcare compared to PLWNCDs from socially advantaged groups. The disadvantaged population (particularly individuals from low socioeconomic conditions) have a high chance of falling sick (23), dying, and experiencing catastrophe. Furthermore, socioeconomically deprived individuals who were dependent on daily wages have lost their jobs; this has pushed them further into poverty and poor health (23).

A synergistic association between the severity of COVID-19 and NCDs was reported in China (24), which shows the negative effects of this syndemic. This suggests the urgency of a paradigm shift from a single-condition approach to a syndemic approach to tackle the current and future impacts of pandemics among PLWNCDs in LMICs. The pandemic is unlikely to end soon, and it is difficult to predict the arrival of the next pandemic, but the syndemic will certainly continue in LMICs. In this paper, we discuss COVID-19 among PLWNCDs, exposure to NCD risk factors, and the disruption of essential public health services for NCDs. It considers literature on this topic, following a search on Google and PubMed to identify publications that considered populations with COVID-19 and NCDs. We conclude by reflecting on strategies and policies that deal with the COVID-19 syndemic among PLWNCDs in LMICs.

## Covid-19 Among PLWNCDs

The global COVID-19 pandemic has resulted in 16,923,006 cases in 213 countries and territories around the world and two international conveyances, with 664,191 fatalities as of July 29, 2020 (25). COVID-19 cases are decreasing in many countries, but the opposite is true in LMICs such as India and Brazil. Many seriously ill COVID-19 patients had multiple comorbidities (26); for instance, 96.2% of those who died in hospitals in Italy had comorbidities. The case fatality rate increases with age, especially in countries with a high percentage of older adults. The comorbidities were mostly NCDs, such as hypertension, diabetes, cardiovascular disease, and chronic lung disease, especially chronic obstructive pulmonary disease (27, 28). The prevalence of comorbidities is higher among COVID-19 patients compared to the general population who are not infected with coronavirus; for instance, 86% of the COVID-19 patients in India and 72% of the COVID-19 patients in China had comorbidities (28). The prevalence of comorbidities is expected to be similar in other LMICs where the prevalence of NCDs is high; however, there is a lack of literature on this topic from LMICs. The health condition is more severe and mortality is higher among older adults with NCDs (29) and people with bacterial infections caused by antibiotic resistant pathogens, such as superinfections (30).

NCDs cause around 72% of deaths worldwide and are the primary cause of death in Southeast Asia among those aged 30 to 70 years (28). LMICs have a large NCD burden; in some LMICs, such as India, there is an early onset of NCDs, thereby increasing the risk of COVID-19 among young individuals (31).

The addition of COVID-19 to pre-existing NCDs results in increased morbidity and mortality (32). NCDs can exhibit several characteristics with infectious manifestations, including parameters like a proinflammatory state and compromised innate immune response (33). This condition is further worsened because many PLWNCDs have been deprived of treatment for their diseases since the onset of the COVID-19 pandemic.

## Covid-19 and Exposure to NCD Risk Factors

Preventive methods for this pandemic, such as physical/social distancing, lockdowns, self-isolation, and quarantine, may increase exposure to NCD risk factors, such as the increased use of tobacco products and alcohol as coping strategies (34), increased reliance on unhealthy processed foods and barriers to physical activities (34), which lead to weight gain (31). These factors increase the incidence of NCDs and related mortality (35). Moreover, financial crises and the lack of social contact might enhance the burden of anxiety and depression among PLWNCDs. The economic slowdown predisposes people to malnourishment, which further increases the risk of infectious diseases (31).

## Disruption of Essential Public Health Services and the Way Forward

Since the COVID-19 pandemic began, prevention and treatment services around the globe have been severely impaired, and the disruption is worse in LMICs. The results from a survey conducted by the WHO in 155 countries (36) revealed that PLWNCDs were not able to access services for their health conditions, which made their lives even more difficult during this crisis. More than 53% of the surveyed countries reported partially or completely impaired services for NCDs and related complications, particularly after the COVID-19 trajectory changed from sporadic to community transmission. This is supported by the stories and pictures of PLWNCDs captured in the news and social media of LMICs, where people were unable to access basic medicines or care (particularly in areas with protracted lockdowns) for their chronic conditions. This problem is exacerbated by the reassignment of health staff from NCD facilities to COVID-19 in all surveyed countries (36) and the disruption of medical supplies and diagnostics as a result of nationwide lockdowns (23). For example, in India, some outpatient services have been temporarily closed, and hospitals have been converted into designated COVID-19 care homes (23). This arrangement will have a further adverse effect on access to healthcare services and treatment adherence by PLWNCDs. Similar painful stories regarding PLWNCDs have been reported in the news and social media platforms of many LMICs, such as Nepal, Bangladesh, Brazil, Pakistan, Ghana, and Iran. Governments in various countries have made efforts to focus on NCD services while tackling COVID-19, but only 42% of low-income countries have done so compared to 72% of high-income countries (HICs) (36). This shows the global impact of COVID-19 on the disruption of healthcare services for NCDs.

The interaction of COVID-19 with other biological and social factors appears to increase the risk of complications, worsen health outcomes, and intensify the burden on healthcare professionals and health systems. On the one hand, there is a global rush to respond to COVID-19 by increasing intensive care unit beds, installing ventilators, extending lockdowns, and adopting other containment measures. On the other hand, there is a disruption of routine health services, such as screening and diagnosis, supplies of essential medicines, and access to health service providers and support services.

The COVID-19 syndemic and other conditions have not only posed a challenge to health systems but have also exposed gaps within the healthcare delivery system in many HICs (e.g., Italy, Spain, and the United States) and LMICS (e.g., Pakistan, India, Nepal, Bangladesh, Mexico, and Brazil). Due to COVID-19, the priorities of health services have shifted; as a result, the progress required to achieve Sustainable Development Goals is threatened (37).

**Figure 2:**
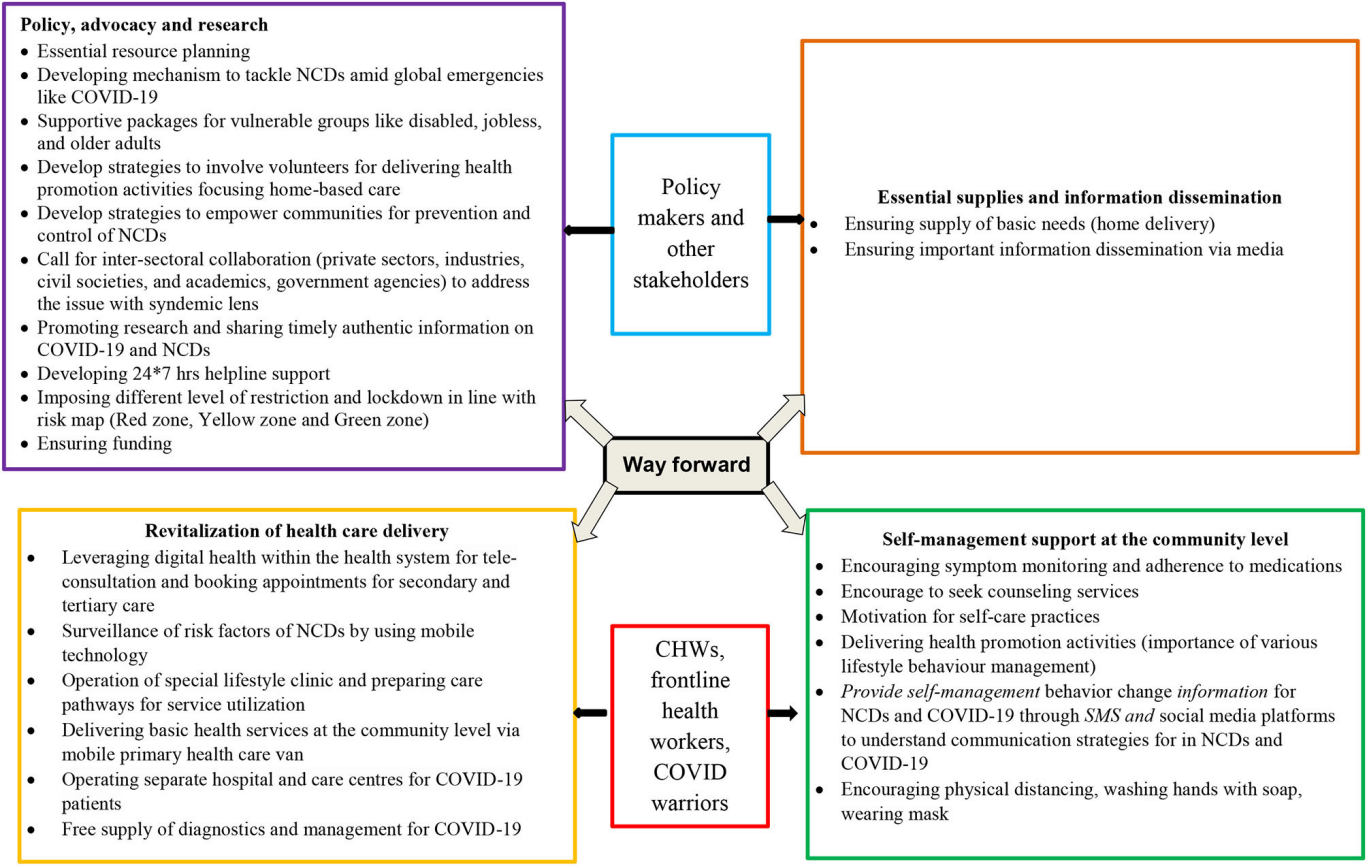
Strategies to overcome and manage the syndemic condition.

In the subsequent section, we describe strategies that are essential to overcoming and managing the syndemic condition. We divide these strategies into four broad categories (Figure 2).

### Essential Supplies and Information Dissemination

The sudden lockdowns imposed by authorities caused panic in many countries. To avoid such situations, there should be a supply of basic needs, such as groceries and sanitary items. Home delivery is an important strategy that can be implemented with the help of volunteers, especially for older adults and people with disabilities. Misinformation and fake news on social media platforms are fuelling this panic. People should follow information from trusted sources such as government guidelines. Additionally, authorities should disseminate the appropriate information to the general public in a timely manner.

### Self-Management Support at the Community Level

PLWNCDs should be encouraged to monitor their symptoms, practice self-care, adhere to medication, seek healthcare services including counseling, practice physical distancing, wash their hands with soap, and wear masks. Providing information on self-management behavior changes for NCDs and COVID-19 through SMS and social media platforms is an important step. In this situation, health literacy (having the necessary information and skills to manage health) and activation (motivation and the ability to take action) can play an important role (37, 38) in self-management (39) of conditions among PLWNCDs in LMICs. Promoting both the health literacy and empowerment of PLWNCDs would enable patients to navigate health services, use technology to contact healthcare providers, develop problem-solving skills, and adhere to healthy lifestyle behaviors (40).

Healthy lifestyle activities must be promoted, such as eating nutritious foods and engaging in physical and wellness activities. Individuals should have access to open spaces and be allowed to exercise at scheduled times while maintaining all precautionary measures, and PLWNCDs could be given timecards for physical activity. The expansion of existing community health worker (CHW) roles can be crucial to the self-management of NCDs and COVID-19 and to delivering basic services among PLWNCDs during this extreme health workforce shortage, particularly in LMICs with weak health systems. Recovered COVID-19 patients can also spread information on health and self-care management and help debunk the myths and lessen the stigma related to COVID-19.

### Revitalizing Healthcare Delivery

Although countries (mostly developed ones) are trying to provide care through telemedicine, it is still in the formative stage. While telemedicine is a boon for developed nations when it comes to the diagnosis, treatment, self-management support, and surveillance of conditions, LMICs with fragile health systems often struggle to launch telemedicine services. Using digital healthcare platforms in the health system (41) would greatly increase access to the services and information required by PLWNCDs. This would, in turn, improve the management of chronic conditions and provide relief from emotional turmoil and stress (42).

In fragmented health systems, CHWs can promote coordinated care by improving access to care and providing navigation support (43). CHWs can also carry out surveillance of risk factors and implement preventive and self-management strategies for PLWNCDs, who are at high risk of COVID-19. Potential CHW roles in COVID-19 management include community engagement, community sensitization, promoting isolation and quarantine, and performing contact tracing (44, 45). Despite their huge potential in pandemic management, CHWs have been underutilized in the COVID-19 pandemic, especially in countries where CHWs are available, such as Bangladesh, India, and Nepal. However, before involving CHWs in the COVID-19 response, they must be provided with appropriate training and adequate personal protective equipment (46).

While responding to COVID-19, the governments of LMICs have failed to ensure health services for PLWNCDs because of the blanket lockdown approach. Insufficient attention has been paid to the unnoticed drivers of COVID-19-related mortality among PLWNCDs. While governments enforce mitigating measures during this pandemic, they also need to develop strategies to map national-level data on NCD patients, as such data do not exist in many LMICs. There is also a need to prepare care pathways for severely ill PLWNCDs by engaging private and public healthcare institutions and delivering basic health services (e.g., screening, medical checkups, and pharmacy services) at the community level *via* mobile primary healthcare vans. In many LMICs, out-of-pocket (OOP) health expenditures are high and will rise further during the COVID-19 pandemic (28). To reduce the burden of OOP due to COVID-19, authorities should make provisions for free diagnostic and treatment facilities and focus on equitable, accessible, and affordable healthcare.

These measures will prevent the deterioration of health among PLWNCDs amid the COVID-19 pandemic.

### Policy, Advocacy, and Research

A situational analysis of available resources and resource planning must be carried out. Supportive packages should be provided to vulnerable groups, such as older adults, people with disabilities, and the unemployed. Involving the private sector, civil society, academia, non-governmental, and governmental organizations through intersectoral coordination and teamwork would address the situation with a syndemic lens. HICs can help LMICs in setting up 24/7 helpline support to provide essential information and guidance related to the availability of services and contact in case of emergency. Authorities should also consider imposing different levels of restrictions by mapping the incidence and active cases of COVID-19, such as by designating red, yellow, and green zones. Providing an uninterrupted supply of funds is a major challenge for LMICs during the COVID-19 pandemic. International organizations, philanthropists, and industrialists through their corporate social responsibility should come forward to help countries facing a financial crisis. High-quality research and data on effective interventions to prevent the spread of infection and treatment of active cases are also needed.

Moreover, authorities should impose taxes on items such as sweetened beverages, tobacco, and alcohol to subsidize prices or lower taxes for nutritious food items and ease movement restrictions for food production, processing, and delivery, which will indirectly lessen the use of unhealthy products.

## Conclusion

COVID-19 and NCDs have a reciprocal effect on each other; NCDs increase vulnerability to COVID-19, and COVID-19 increases NCD-related risk factors. The COVID-19 pandemic may not be the last to threaten the global community. Therefore, there is a need to understand the drivers of the syndemic and design safety nets. The health system must address not just one or some medical problems but ensure holistic care for those that need it, particularly PLWNCDs. Care for PLWNCDs, who are at most risk of COVID-19, must be included in national response frameworks and plans so that the government can protect citizens' health and well-being during the current COVID-19 pandemic and for similar crises in the future, otherwise, the interaction of COVID-19 and NCDs will result in disastrous effects that could be difficult to handle given the preexisting stress on healthcare delivery systems and impede progress in achieving the Sustainable Development Goals.

The governments of LMICs are crippled by a lack of technical and financial resources to address this overwhelming problem. Tackling the COVID-19 syndemic is a matter of urgency. Funding bodies that advocate for and want to be part of a change in LMICs need to invest in prevention and health promotion programs that could address issues within a syndemic framework (47). Government agencies positioned to develop and implement policies must understand that asking citizens to sacrifice without providing appropriate support packages will not work. Rather than gearing up for a vertical approach, governments, concerned stakeholders, development partners, and civil society must build synergy across healthcare platforms to tackle this crisis through a holistic approach. If they fail to do so, the post-pandemic era could experience a great divide in health equity that could be much worse than ever before, undoing the progress made in developing healthcare policies and strengthening healthcare systems and infrastructure. Evidence-guided decisions must be made to overcome this formidable crisis in LMICs.

## Data Availability Statement

The original contributions presented in the study are included in the article/supplementary material, further inquiries can be directed to the corresponding author/s.

## Author Contributions

UNY conceived the idea. UNY, BR, and SKY drafted the manuscript. SP and SKM provided the significant inputs. All authors approved the final version of manuscript.

## Conflict of Interest

The authors declare that the research was conducted in the absence of any commercial or financial relationships that could be construed as a potential conflict of interest.

## References

[1] Sobral MFF Duarte GB da Penha Sobral AIG Marinho MLM de Souza Melo A. Association between climate variables and global transmission oF SARS-CoV-2. Sci Total Environ. (2020) 729:138997. 10.1016/j.scitotenv.2020.138997 32353724 PMC7195330

[2] OECD The World Economy on a Tightrope France: Organisation for Economic Co-operation and Development. (2020). Available online at: http://oecd.org/economic-outlook

[3] Galea S Merchant RM Lurie N. The mental health consequences of COVID-19 and physical distancing: the need for prevention and early intervention. JAMA Intern Med. (2020) 180:817–8. 10.1001/jamainternmed.2020.1562 32275292

[4] Holmes EA O'Connor RC Perry VH Tracey I Wessely S Arseneault L. Multidisciplinary research priorities for the COVID-19 pandemic: a call for action for mental health science. Lancet Psychiatry. (2020) 7:547–60. 10.1016/S2215-0366(20)30168-1 32304649 PMC7159850

[5] Duan L Zhu G. Psychological interventions for people affected by the COVID-19 epidemic. Lancet Psychiatry. (2020) 7:300–2. 10.1016/S2215-0366(20)30073-0 32085840 PMC7128328

[6] Thakur V Jain A. COVID 2019-suicides: a global psychological pandemic. Brain Behav Immun. (2020) 88:952–3. 10.1016/j.bbi.2020.04.062 32335196 PMC7177120

[7] Hawryluck L Gold WL Robinson S Pogorski S Galea S Styra R SARS control and psychological effects of quarantine, Toronto, Canada. Emerg Infect Dis. (2004) 10:1206–12. 10.3201/eid1007.030703 15324539 PMC3323345

[8] Singer M A dose of drugs, a touch of violence, a case of AIDS: conceptualizing the SAVA syndemic. Free Inq Creativ Sociol. (1996). 24:99–110.

[9] Bambra C Riordan R Ford J Matthews F The COVID-19 pandemic and health inequalities. J Epi Commu Health. (2020) 1–5. 10.1136/jech-2020-214401 PMC7298201 32535550

[10] Shiau S Krause KD Valera P Swaminathan S Halkitis PN. The burden of COVID-19 in people living with HIV: a syndemic perspective. AIDS Behav. (2020) 24:2244–9. 10.1007/s10461-020-02871-9 32303925 PMC7165075

[11] Brown E Gray R Lo Monaco S O'Donoghue B Nelson B Thompson A. The potential impact of COVID-19 on psychosis: a rapid review of contemporary epidemic and pandemic research. Schizophr Res. (2020) 1–9. 10.1016/j.schres.2020.05.005 32389615 PMC7200363

[12] Dubey S Biswas P Ghosh R Chatterjee S Dubey MJ Chatterjee S Psychosocial impact of COVID-19. Diabetes Metab Syndr Clin Res Rev. (2020) 14:779–88. 10.1016/j.dsx.2020.05.035 PMC7255207 32526627

[13] Yadav UN Tamang MK Thapa TB Hosseinzadeh H Harris MF Yadav KK. Prevalence and determinants of frailty in the absence of disability among older population: a cross sectional study from rural communities in Nepal. BMC Geriatr. (2019) 19:283. 10.1186/s12877-019-1290-0 31640571 PMC6806560

[14] Siriwardhana DD Hardoon S Rait G Weerasinghe MC Walters KR. Prevalence of frailty and prefrailty among community-dwelling older adults in low-income and middle-income countries: a systematic review and meta-analysis. BMC J. (2018). 8:e018195. 10.1136/bmjopen-2017-018195 29496895 PMC5855322

[15] Branca F Lartey A Oenema S Aguayo V Stordalen GA Richardson R. Transforming the food system to fight non-communicable diseases. BMJ. (2019) 364:l296. 10.1136/bmj.l296 30692128 PMC6349221

[16] Yadav UN Thapa TB Mistry SK Pokhrel R Harris MF. Socio-demographic characteristics, lifestyle factors, multi-morbid conditions and depressive symptoms among Nepalese older adults. BMC Psychiatry. (2020) 20:261. 10.1186/s12888-020-02680-3 32456611 PMC7249669

[17] Remais JV Zeng G Li G Tian L Engelgau MM. Convergence of non-communicable and infectious diseases in low- and middle-income countries. Int J Epidemiol. (2012) 42:221–7. 10.1093/ije/dys135 23064501 PMC3600620

[18] Wang B Li R Lu Z Huang Y. Does comorbidity increase the risk of patients with COVID-19: evidence from meta-analysis. Aging. (2020) 12:6049–57. 10.18632/aging.103000 32267833 PMC7185114

[19] Gunnell D Appleby L Arensman E Hawton K John A Kapur N. Suicide risk and prevention during the COVID-19 pandemic. Lancet Psychiatry. (2020) 7:468–71. 10.1016/S2215-0366(20)30171-1 32330430 PMC7173821

[20] Mamun MA Ullah I. COVID-19 suicides in Pakistan, dying off not COVID-19 fear but poverty?—the forthcoming economic challenges for a developing country. Brain Behav Immu. (2020) 87:163–6. 10.1016/j.bbi.2020.05.028 32407859 PMC7212955

[21] Kretchy IA Asiedu-Danso M Kretchy JP. Medication management and adherence during the COVID-19 pandemic: perspectives and experiences from low-and middle-income countries. Res Soc Adm Pharm. (2020) 1–4. 10.1016/j.sapharm.2020.04.007 32307319 PMC7158799

[22] Banerjee M Chakraborty S Pal R. Diabetes self-management amid COVID-19 pandemic. Diabetes Metab Syndr. (2020) 14:351–4. 10.1016/j.dsx.2020.04.013 32311652 PMC7194953

[23] Basu S. Non-communicable disease management in vulnerable patients during Covid-19. Indian J Med Ethics. (2020) V:103–5. 10.20529/IJME.2020.041 32393447

[24] Epidemiology Working Group for NCIP Epidemic Response Chinese Center for Disease Control and Prevention. [The epidemiological characteristics of an outbreak of 2019 novel coronavirus diseases (COVID-19) in China]. Zhonghua Liu Xing Bing Xue Za Zhi. (2020) 41:145–51. 10.3760/cma.j.issn.0254-6450.2020.02.003 32064853

[25] Covid-19 Coronavirus Pandemic (2020). Available online at: https://www.worldometers.info/coronavirus/ (accessed June 13, 2020).

[26] Pal R Bhadada SK. COVID-19 and non-communicable diseases. BMJ J. (2020) 96:429–30. 10.1136/postgradmedj-2020-137742 32234837 PMC10016830

[27] Kluge HHP Wickramasinghe K Rippin HL Mendes R Peters DH Kontsevaya A. Prevention and control of non-communicable diseases in the COVID-19 response. Lancet. (2020) 395:1678–80. 10.1016/S0140-6736(20)31067-9 32401713 PMC7211494

[28] Thakur J Novel coronavirus pandemic may worsen existing global noncommunicable disease crisis. Int J Noncommun Dis. (2020) 5:1–3. 10.4103/jncd.jncd_2_20

[29] HHP K Older People are at Highest Risk From COVID-19, but all Must act to Prevent Community Spread Copenhegan: World Health Organisation Reginal Office for Europe. (2020). Available online at: https://www.euro.who.int/en/health-topics/health-emergencies/coronavirus-covid-19/statements/statement-older-people-are-at-highest-risk-from-covid-19,-but-all-must-act-to-prevent-community-spread (accessed June 4, 2020).

[30] Clancy CJ Nguyen MH. Coronavirus disease 2019 superinfections, and antimicrobial development: what can we expect? Clin Infect Dis. (2020) ciaa524. 10.1093/cid/ciaa524 32361747 PMC7197597

[31] Gopalan HS Misra A. COVID-19 pandemic and challenges for socio-economic issues, healthcare and national health programs in India. Diabetes Metab Syndr. (2020) 14:757–9. 10.1016/j.dsx.2020.05.041 32504992 PMC7261093

[32] World Heart Federeation. COVID 19 and CVD: World Heart Federation. (2020). Available online at: https://www.world-heart-federation.org/covid-19-and-cvd/ (accessed June 4, 2020).

[33] Chen L Deng H Cui H Fang J Zuo Z Deng J. Inflammatory responses and inflammation-associated diseases in organs. Oncotarget. (2018) 9:7204–18. 10.18632/oncotarget.23208 29467962 PMC5805548

[34] NCD Alliance Impacts of COVID-19 on people living with NCDs. NCD Alliance (2020). Available online at: https://ncdalliance.org/sites/default/files/resource_files/COVID-19_%26_NCDs_BriefingNote_27April_FinalVersion_0.pdf (accessed June 10, 2020).

[35] Alqahtani JS Oyelade T Aldhahir AM Alghamdi SM Almehmadi M Alqahtani AS. Prevalence, severity and mortality associated with COPD and smoking in patients with COVID-19: a rapid systematic review and meta-analysis. PLoS ONE. (2020) 15:e0233147. 10.1371/journal.pone.0233147 32392262 PMC7213702

[36] WHO. COVID-19 Significantly Impacts Health Services for Noncommunicable Diseases: World Health Organisation. (2020). Available online at: https://www.who.int/news-room/detail/01-06-2020-covid-19-significantly-impacts-health-services-for-noncommunicable-diseases (accessed June 4, 2020).

[37] Khetrapal S Bhatia R. Impact of COVID-19 pandemic on health system & Sustainable Development Goal 3. Indian J Med Res. (2020) 151:395–9. 10.4103/ijmr.IJMR_1920_20 32611910 PMC7530436

[38] Hearn J Ssinabulya I Schwartz JI Akiteng AR Ross HJ Cafazzo JA. Self-management of non-communicable diseases in low- and middle-income countries: A scoping review. PLoS ONE. (2019) 14:e0219141. 10.1371/journal.pone.0219141 31269070 PMC6608949

[39] Yadav UN Hosseinzadeh H Lloyd J Harris MF. How health literacy and patient activation play their own unique role in self-management of chronic obstructive pulmonary disease (COPD)? Chron Respir Dis. (2019) 16:1479973118816418. 10.1177/1479973118816418 30789021 PMC6318723

[40] Paakkari L Okan O. COVID-19: health literacy is an underestimated problem. Lancet Public Health. (2020) 5:e249–50. 10.1016/S2468-2667(20)30086-4 32302535 PMC7156243

[41] Zarocostas J. How to fight an infodemic. Lancet. (2020) 395:676. 10.1016/S0140-6736(20)30461-X 32113495 PMC7133615

[42] Mahmood S Hasan K Colder Carras M Labrique A. Global preparedness against COVID-19: we must leverage the power of digital health. JMIR Public Health Surveill. (2020) 6:e18980. 10.2196/18980 32297868 PMC7164944

[43] Balaban RB Galbraith AA Burns ME Vialle-Valentin CE Larochelle MR Ross-Degnan D. A patient navigator intervention to reduce hospital readmissions among high-risk safety-net patients: a randomized controlled trial. J Gener Intern Med. (2015) 30:907–15. 10.1007/s11606-015-3185-x 25617166 PMC4471016

[44] Bhaumik S Moola S Tyagi J Nambiar D Kakoti M. Community health workers for pandemic response: a rapid evidence synthesis. BMJ Global Health. (2020) 5:e002769. 10.1136/bmjgh-2020-002769 32522738 PMC7292038

[45] Ballard M Bancroft E Nesbit J Johnson A Holeman I Foth J. Prioritising the role of community health workers in the COVID-19 response. BMJ Global Health. (2020) 5:e002550. 10.1136/bmjgh-2020-002550 32503889 PMC7298684

[46] McMahon SA Ho LS Brown H Miller L Ansumana R Kennedy CE. Healthcare providers on the frontlines: a qualitative investigation of the social and emotional impact of delivering health services during Sierra Leone's Ebola epidemic. Health Policy Plan. (2016) 31:1232–9. 10.1093/heapol/czw055 27277598 PMC5035780

[47] Singer M Clair S. Syndemics and public health: reconceptualizing disease in bio-social context. Med Anthropol Q. (2003) 17:423–41. 10.1525/maq.2003.17.4.423 14716917

